# ERP Correlates of Encoding Success and Encoding Selectivity in Attention Switching

**DOI:** 10.1371/journal.pone.0167396

**Published:** 2016-12-01

**Authors:** Franziska R. Richter, Nick Yeung

**Affiliations:** Department of Experimental Psychology, University of Oxford, Oxford, United Kingdom; Kessler Foundation, UNITED STATES

## Abstract

Long-term memory encoding depends critically on effective processing of incoming information. The degree to which participants engage in effective encoding can be indexed in electroencephalographic (EEG) data by studying event-related potential (ERP) subsequent memory effects. The current study investigated ERP correlates of memory success operationalised with two different measures—memory selectivity and global memory—to assess whether previously observed ERP subsequent memory effects reflect focused encoding of task-relevant information (memory selectivity), general encoding success (global memory), or both. Building on previous work, the present study combined an attention switching paradigm—in which participants were presented with compound object-word stimuli and switched between attending to the object or the word across trials—with a later recognition memory test for those stimuli, while recording their EEG. Our results provided clear evidence that subsequent memory effects resulted from selective attentional focusing and effective top-down control (memory selectivity) in contrast to more general encoding success effects (global memory). Further analyses addressed the question of whether successful encoding depended on similar control mechanisms to those involved in attention switching. Interestingly, differences in the ERP correlates of attention switching and successful encoding, particularly during the poststimulus period, indicated that variability in encoding success occurred independently of prestimulus demands for top-down cognitive control. These results suggest that while effects of selective attention and selective encoding co-occur behaviourally their ERP correlates are at least partly dissociable.

## Introduction

A founding observation in research on human memory is that successful encoding depends on establishing an effective cognitive ‘set’ for processing incoming information: Memory will improve to the extent that presented information is attended [1], organised [2], and processed deeply [3], ideally in a manner that relates meaningfully to the way information will be retrieved later [4]. The degree to which participants engage in effective encoding—that is, adopt an appropriate encoding set—can be robustly measured in the scalp-recorded electroencephalogram (EEG) in terms of *subsequent memory effects*, neural processing differences that are predictive of later memory [5].

The present study focuses on event-related potential (ERP) subsequent memory effects to ask two related questions. The first is whether subsequent memory effects reflect global readiness to encode any available information into memory, a selective cognitive set for encoding a particular type of information [6,7], or a combination of both factors. The second question is whether successful encoding depends on similar mechanisms of top-down cognitive control to those underpinning successful attention switching or task switching [8,9], which would indicate shared cognitive mechanisms (cf. [10]). To address these questions, we combined an attention switching design—in which participants saw compound object-word stimuli on each trial, and switched between attending to the object or the word across trials—with a later recognition memory test for those stimuli. Critically, the attention-switching phase serves as an incidental encoding task, giving a manipulation of how attention is allocated during encoding.

Previous ERP studies have identified several subsequent memory effects, measured as activity differences that predict whether stimuli are later remembered or forgotten. Subsequent memory effects were first identified in stimulus-locked potentials—that is, ERPs observed following presentation of to-be-remembered material [5,11]. Such effects are commonly observed as differences in early potentials such as the P3 as well as in positive slow waves over fronto-central or centro-parietal electrodes that emerge a few hundred milliseconds after stimulus onset [11,12]. Slow-wave effects are typically more frontally distributed if elaborate or deep encoding strategies are used (e.g., [13,14]), and are weaker and more centro-parietally distributed for rote encoding (e.g., [15]). In contrast, more centro-parietal effects have been linked to rote or perceptual encoding [11,17].

Since first being described, post-stimulus subsequent memory effects have been consistently reported, and have provided valuable insight into the mechanisms of long-term memory formation [18,19]. However, the typical design of previous studies—in which a single, task-relevant stimulus is presented on each encoding trial—leaves open a crucial question: whether subsequent memory effects reflect global enhancement of mnemonic processing, for example because the current neural state is particularly conducive to encoding (e.g., [18]), whether they reflect selective focussing on task-relevant material that leads to effective encoding of this material alone, or whether they can be reflective of both. For example, the observation of frontally-focused subsequent memory effects during controlled or elaborative encoding [14] could indicate an overall increase in alertness that is beneficial for later memory (cf. [20]) or the formation of a selective encoding set that facilitates semantic processing.

The first aim of the present study was therefore to contrast global and selective contributions to subsequent memory effects. To this end, our experiment built on previous studies in which we combined task switching with a later recognition memory test. Participants first switched between object and word classification tasks, performed on picture–word stimuli that each appeared only once, and were later tested for their recognition memory of these items separately. As in previous studies, we measure *memory selectivity* in terms of increased recognition confidence for task-relevant over task-irrelevant information. In this study we introduce a new measure that we term *global memory*, an index of general encoding success for items regardless of their task relevance (i.e., increased recognition confidence for task-relevant and irrelevant information combined). In a small methodological departure from our previous studies, participants performed the same natural/human-made classification on every trial, and only switched across trials whether this judgment was applied to the object or word presented (rather than switching both the attended item and the required semantic judgment). We made this change to deconfound switches of attention from switches in the semantic task, thereby simplifying the interpretation of any observed differences in neural activity. This change additionally allowed us to test whether our previous results [21–23] would replicate when only attention was switched, as one would expect given previous work suggesting that common neural mechanisms might underlie switching both attention and task [24]. Regardless, the present study shared the crucial feature of our past work that participants were presented in each trial with both task-relevant (attended) and task-irrelevant (unattended) information, allowing us to test for differences in processing of both types of information.

On the basis of prior work showing that task switching affects memory primarily in terms of changes in selectivity [21], we predicted that subsequent memory effects would track changes in encoding selectivity. Including the additional variable of global memory provides the opportunity to test for general encoding effects on memory, such as the overall cognitive demand associated with switching attention. A critical assumption here is that later memory serves as “a window onto the architecture of cognitive control processes” in the preceding incidental encoding phase (cf. [25], p. 11944). Consistent with this assumption, later memory has been shown to be a good indicator of the effectiveness of attention during earlier task switching, as reflected in a negative relationship between task-switching RTs and selectivity of encoding [21]. Extending beyond this behavioural analysis, here we investigated ERP correlates of memory-related processes during task switching.

Although we were primarily interested in post-stimulus subsequent memory effects, we also analyzed pre-stimulus preparatory ERP components. Recent research suggests that successful memory can be predicted from preparatory EEG activity seen in the period before stimulus onset [7,26–28]. Although these prestimulus effects seem to be somewhat more elusive than post-stimulus subsequent memory effects, studies of prestimulus effects have provided evidence of both global mnemonic benefits as well as material-selective subsequent memory effects: Whereas some studies observe similar preparatory effects regardless of stimulus type (e.g., visual and auditory words; [27], suggestive of global effects), other studies report prestimulus effects that exhibit different topographies depending on the encoding task (e.g., [7,28]) or stimulus type (e.g., visual versus auditory words, [6]), consistent with selective effects.

Our second aim was to contrast the ERP correlates of memory encoding and attention switching. In previous work we have used behavioural measures to demonstrate a relationship between memory and switching. Thus, just as performance is impaired during switching because processing of task-relevant information is disrupted by the presence of distracting task-irrelevant information [29–31], switching affects later memory in terms of impaired memory for task-relevant items but surprisingly improved memory for task-irrelevant items [21]. Meanwhile, factors that facilitate effective top-down control, such as increased preparation time and reward incentives, lead to improved switching and corresponding increases in the selectivity of memory (cf. [10,22]). These behavioural results suggest shared mechanisms of cognitive control in task switching and memory, which establish strong task sets to enable fast and accurate responding to task-relevant stimuli, and correspondingly lead to good memory for those stimuli (and poor memory for task-irrelevant items).

Behavioural measures therefore suggest a close relationship between cognitive control and long-term memory, a conclusion consistent with a growing corpus of neuroimaging findings [10,20,23]. However, although some very recent studies have combined ERP with attention switching and later memory [32,33], it remains unclear whether there is meaningful overlap in the neural correlates. For example, whereas attention switching primarily modulates decision-related components such as the N2 and P3 that are tightly time-locked to participants’ responses to presented stimuli [34], stimulus-locked subsequent memory effects are additionally observed in distinct late slow waves whose topography varies according to the nature of the encoding task (e.g., [35]). It is difficult, though, to draw strong conclusions from studies with divergent methodologies and different encoding tasks. Our second aim was therefore to provide a systematic comparison of the ERP correlates of attention switching and successful memory encoding in a single study.

Although the main focus of this paper is on stimulus-locked effects, we also present cue-locked analyses with a more exploratory flavor, as prestimulus attention switching effects and subsequent memory effect show at least superficial similarities. For example, EEG activity observed during preparation for an upcoming switch is characterized by frontal negative slow waves (e.g., [36,37]) and an accompanying posterior positivity [37–40], reminiscent of the prestimulus subsequent memory effect reported in some memory studies (e.g., [7]). Moreover, both forms of preparatory activity exhibit task dependency, being selectively observed prior to difficult switches [37] and deep encoding tasks [7].

In summary, we analysed pre- and post-stimulus ERP data in an object-word attention-switching paradigm to probe the relationship between memory encoding and top-down control. Our first aim was to determine whether ERP subsequent memory differences are primarily observed in relation to the selectivity of memory for task-relevant items (thus indicating a selective encoding effect), in relation to the global amount of information encoded regardless of task relevance (suggesting that they track more general encoding processes), or a combination of both. Our second aim was to compare these subsequent memory effects to established ERP markers of attention switching, to test whether ERP measures would show similar co-dependencies as have been observed in our previous behavioural work [22].

## Methods

### Participants

There were eight male and eight female participants, all right-handed native English speakers, with a mean age of 21.9 years (*SD* = 4.1). All gave written informed consent, which included confirmation that they understood the procedures and were free to withdraw from the experiment at any time without any negative consequences. They received payment or course credit for participation. Ethical approval was obtained from the University of Oxford Research Ethics Committee for research involving human participants (OXTREC 53 08).

### Material

**Fig 1:**
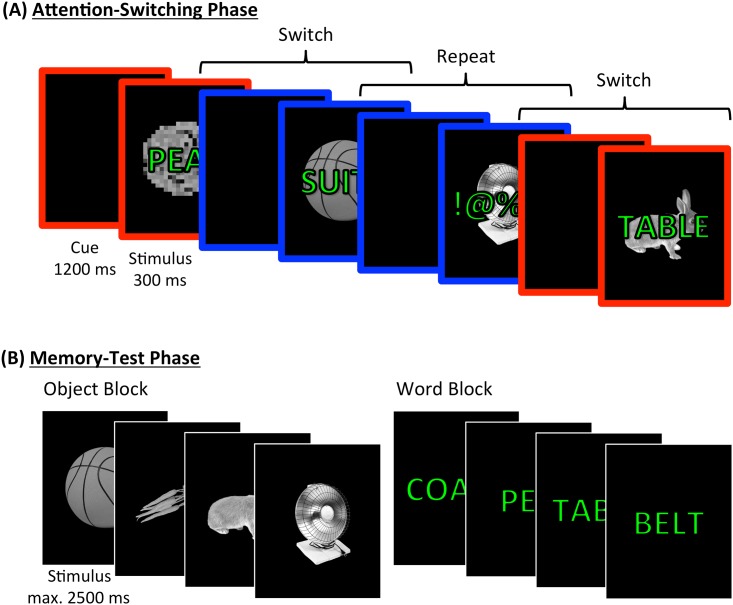
**A:** Simplified example of trials in the attention-switching phase. Each trial began with a coloured frame indicating the required task (e.g., red = word classification, blue = object classification, with the colour counterbalanced across participants). Following the cue, a compound stimulus was presented for 300 ms. On univalent trials, the stimulus consisted either of an object and a random character string or of a word and a scrambled object (see first and third stimulus in Fig 1A). On bivalent trials, the stimulus consisted of a picture of an object and a word (see second and fourth stimulus in Fig 1A). Each trial was followed by an empty cue frame until the participant’s response, after which there was an intertrial interval (500 ms, both not shown). **Fig 1B**: The memory test consisted of blocks in which only object pictures were probed (‘Object Blocks’, left section of Fig 1B) and blocks in which only word stimuli were probed (‘Word Blocks’, right section of Fig 1B). Participants had a maximum of 2500 ms to respond, before the program moved on. If no response was given, a warning (“LATE!”, not shown) appeared for 1000 ms before the next trial started.

Stimuli were words, object pictures, random character strings, and scrambled object pictures. Stimuli were presented on a black background with words/strings superimposed on the pictures in green font. The pictures were photo-realistic objects from the Hemera Photo-Objects Collections (Hemera Photo Objects, Hull, Quebec, Canada). The words were a subset taken from the stimulus set reported by Poldrack et al. [41], comprising nouns that were 3–10 letters (*M* = 6.02, *SD* = 1.44) and 1–3 syllables long. To create scrambled object pictures, each original object picture was divided into squares that were then randomly interchanged, after which RGB values within each square were averaged across pixels (for an example see Fig 1A). Random character strings were constructed by replacing letters in experimental words with non-alphanumeric characters (e.g.,!, £,%,§, =, # etc.). Words and objects were assigned randomly to the experimental conditions, separately for each participant.

### Procedure

The current study employed an attention switching paradigm—which critically also served as an encoding phase—combined with a subsequent memory test. In the attention-switching phase, participants switched between making natural/human-made judgments on object pictures or word stimuli. Trials were classified as repeat trials if the same material was attended in the previous trial and as switch trials if different material was attended. Following the attention switching phase, participants completed a recognition memory test that included items presented during the attention-switching phase, together with new items.

On most attention-switching trials (2/3, i.e., 200 trials), the task-relevant object or word was presented together with an item from the task-irrelevant category (word or object, respectively; 100 trials each). These *bivalent* trials provide the critical test of attention and memory for task-relevant and task-irrelevant information. In addition, 1/3 (i.e., 100 trials) of trials were *univalent*, in that the natural/human-made categorization task could only be performed on the task-relevant information (i.e., the task could not be performed on scrambled pictures or meaningless character strings). During the attention-switching phase, the same classification rule was used for both material types: a judgement of whether the to be attended word or object was natural and human-made.

The currently-relevant material type was cued by a coloured frame appearing with a 1200 ms cue-stimulus interval (CSI). Red and blue frames were used, with the mapping of cue-colour to task counterbalanced between participants. After the CSI, the compound stimulus was presented for 300 ms before disappearing. The cue remained on the screen until the participant’s response, after which there followed a 500 ms inter-trial interval (ITI). Participants completed 6 attention-switching blocks of 50 trials each, in which switch and repeat trials were equally frequent. Participants responded with their index fingers using the ―”x” (human-made) and ―”n” (natural) keys for both material types. The word and picture stimuli did not overlap (e.g., the word “ball” would not occur if a picture of a ball was used).

The following recognition memory test comprised 8 blocks of 74 trials in which The ratio of old to new items was 5:1. Blocks contained only objects (O blocks) or only words (W blocks), delivered in WOOWWOOW or OWWOOWWO order, counterbalanced across participants (see also Fig 1B). Stimuli were presented in randomised order within the blocks. Participants rated each stimulus on a 1–6 scale, with 1 indicating a sure judgment that the item was new and 6 indicating a sure judgment that the item was old.

Participants were instructed to use the 6 response only when they were able to recall specific detail about having seen this item in the attention-switching phase. Ratings between extreme values of 1 and 6 were used for less confident answers. After each block participants received feedback on their performance. Answers to the memory test were time limited to 2500 ms, otherwise the word “LATE!” appeared in red letters for 1000 ms. The ITI was 500 ms. EEG was recorded from the beginning of the attention-switching phase to the end of the memory test.

### Behavioural data analysis

Analysis of attention-switching performance excluded post-error trials as well as reaction time (RT) outliers (3 *SD* above the mean, separately for each task, switch vs. repeat trials, uni vs. bivalent trials, and for each participant; using this cut off 2.4% of switch trials and 2.05% of repeat trials were excluded as outliers). In analyses of recognition memory performance, trials answered incorrectly during attention switching were excluded. We take participants’ recognition memory ratings as an index of the quality of retrieved memories—in common with much prior research [42], where subjective ratings are typically found to be predictive of objective accuracy [43,44]—while acknowledging that higher confidence is an imperfect index of better memory (e.g., [45,46]).

To investigate selective and global encoding success we computed two different measures. We calculated the degree to which participants more confidently recognized task-relevant than task-irrelevant items from each bivalent attention-switching trial as a trial-by-trial measure of memory selectivity [21]. The resulting score thus ranged from +5 (if the attended item was rated 6—“sure old”, and the unattended item rated 1—“sure new”) to -5 (if the opposite ratings were given). We additionally calculated “global memory” as a trial-by-trial measure of overall encoding success—the degree to which participants confidently recognize items regardless of task relevance. Global memory scores are calculated here for each bivalent attention-switching trial by summing memory ratings for attended and unattended items. The resulting score thus ranges from +12 (if both attended and unattended items were rated 6—“sure old) to +2 (if both items were rated as “sure new”). Once these scores were calculated, trials were divided into three bins according to each score, separately for each participant and for switch and repeat trials. The cut-off value for each bin was determined such that scores were divided into maximally balanced bins using Matlab’s ‘tiedrank’ function, ensuring that values of the same kind would be grouped together rather than being split across groups.

### EEG recording and analysis

Participants sat in a comfortable chair in an electrically shielded room. EEG data were collected with SynAmps2 amplifiers (Neuroscan, El Paso, TX), from 32 Ag/AgCl electrodes embedded in a fabric cap at locations FP1, FPZ, FP2, F7, F3, FZ, F4, F8, FT7, FC3, FCZ, FC4, FT8, T7, C3, CZ, C4, T8, TP7, CP3, CPZ, CP4, TP8, P7, P3, PZ, P4, P8, POZ, O1, OZ, and O2. Two electrodes were attached on the outer canthi of both eyes, and another two electrodes were attached above and below the left eye to record blinks and eye movement activity. Electrodes were furthermore attached to the left and right mastoids, with the right mastoid serving as the reference electrode. No re-referencing was applied offline. The impedances of all electrodes were kept below 50 kΩ. A sampling rate of 1000 Hz, and an online high-pass filter of 0.1 Hz were used for the recording of the EEG data. Ocular artefact correction was conducted in Neuroscan using a regression approach [47]. Before analysis, the EEG data were downsampled to 100 Hz.

EEG data analysis focussed on the attention-switching phase because we were interested in ERP correlates of successful task-performance and encoding. Accordingly, the continuous data were segmented from −500 ms to 2300 ms relative to experimental events of interest, time-locked to the presentation of the cue (cue ERPs) or the stimulus (stimulus ERPs). Epochs were baseline corrected by subtracting the average activity of each channel during the -100 to 0 ms period prior to cue or stimulus onset, respectively. Trials were rejected if at least one electrode showed a difference of more than 150 μV from the beginning to the end of the predefined time window (0–1200 ms for cue ERPs, 0–2300 ms for stimulus ERPs). A low-pass filter of 20 Hz was used for analysis using the “eegfilt” method implemented in the EEGLab toolbox. All further ERP analyses described below were completed in MATLAB using the EEGLab toolbox and custom-written routines.

Two participants were excluded from the ERP analysis because insufficient trial numbers were preserved for analysis after the artefact rejection, leaving 14 participants for the ERP analysis. In the cue-locked phase, the lowest number of trials included for a participant was 187 out of 300 trials (all other participants had more than 200 trials); in the stimulus-locked phase, due to the longer interval, the lowest number of trials included for a participant was 141 out of 300 trials (one other participant had 171 trials; all other participants had more than 200 trials).

Due to the aforementioned suggestions of a dissociation between frontal and posterior subsequent memory effects, our analysis focused on anterior-posterior differences in topography. Specifically, electrodes were divided into two equal-sized clusters over frontal and posterior electrode sites, and activity was averaged across electrodes in these clusters. The frontal cluster contained electrodes F3, FZ, F4, FC3, FCZ, and FC4; the posterior cluster contained electrodes CP3, CPZ, CP4, P3, PZ, and P4. The ERP data were furthermore divided into 300 ms time windows (with the ERP amplitude averaged across each window), as it was expected that switch- and memory-related preparation- and stimulus-related effects would develop over time, and might differ for memory selectivity and global memory measures. The window length of 300 ms was chosen after visual inspection of the across-condition ERP (i.e., before the data was split into the different experimental conditions of interest). This ERP showed both early visual (0–300) as well as later slow wave potentials. Memory selectivity and global memory were analysed separately as it is problematic to compare them directly since they are based on the same data (memory ratings for attended and unattended items), just being computed in different ways (subtraction of unattended from attended scores for memory selectivity vs. addition of scores for global memory). Memory selectivity and global memory scores were split into two bins only (rather than three bins, as was done in the behavioural data) because fewer trials were available in this ERP analysis after artefact rejection.

We had included univalent trials in this experiment because contrasting the two stimulus conditions might give a relatively pure measure of object- and word-related activity in the stimulus phase, in contrast to bivalent trials in which both an object and a word were presented simultaneously. Univalent trials did not reveal consistent material-specific effects in a first exploratory analysis and were therefore not included in the analyses here, because our hypotheses were crucially concerned with memory ratings for both task-relevant and task-irrelevant items.

Where the assumption of sphericity was violated in reported ANOVAs, *p*-values based on adjusted degrees of freedom according to Greenhouse-Geisser will be reported with original *F*-values and original degrees of freedom, alongside the Greenhouse-Geisser Epsilon value.

## Results

### Behavioural results

#### Attention switching

Analyses of attention-switching performance focused on the effects of switching on univalent and bivalent trials. In an ANOVA including variables Mode (univalent vs. bivalent) and Switch (switch vs. repeat), the main effects of Switch, *F*(1, 15) = 29.92, *p* < .01, (repeat = 958 ms vs. switch = 1171 ms), and Mode, *F*(1, 15) = 7.39, *p* < .05, (univalent = 1045 ms vs. bivalent = 1083 ms) were significant. Thus, we observed switch costs (slower responses in switch than repeat trials), and bivalent trials showed increased RTs compared to univalent trials. There was no reliable interaction between Mode and Switch in the RT data, *F* < 1, and no significant effects at all were found in the error rates, all *Fs* < 1, with error rates overall being low (mean error rates bivalent 4.2% vs. univalent 3.5%, and switch 4.0% vs. repeat 3.7%) relative to our previous study [21] in which a shorter CSI was used, and participants switched both the semantic task and the material attended. Thus, our RT data displayed the expected costs of switching, and of responding to bivalent as compared to univalent stimuli.

### Memory

#### Effects of switching and attention on Memory

Analysis of the memory data focused on recognition memory ratings for items appearing on bivalent attention-switching trials because only in these trials there was both an attended and an unattended item. Memory ratings were significantly higher for attended items (*M* = 4.46, *SE* = 0.10) than for unattended items (*M* = 3.14, *SE* = 0.08), *t*(15) = 12.76, *p* < .01, but participants still rated unattended items reliably higher than new items (*M* = 2.68, *SE* = 0.07), *t*(15) = 6.79, *p* < .01.

**Fig 2:**
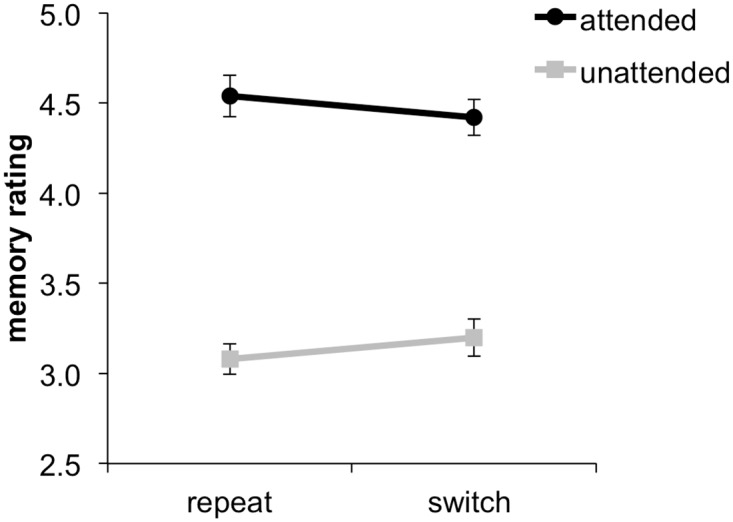
Memory ratings for attended and unattended items presented during repeat and switch trials. Error bars indicate standard errors of the mean.

A first analysis focussed on the crucial relationship between attention and memory encoding. Replicating previous findings [21,22], an ANOVA on mean memory ratings with variables Switch and Attention (attended vs. unattended) revealed a main effect of Attention, *F*(1, 15) = 173.30, *p* < .01, with higher memory ratings for attended than unattended items (mean memory ratings: 4.48 vs. 3.14), no significant main effect of Switch, *F* < 1 (mean memory ratings repeat: 3.81 vs. switch 3.81), but a significant interaction between Switch and Attention, *F*(1, 15) = 5.00, *p* < .05, that revealed the predicted reduction in memory selectivity with attention switching (see Fig 2). Thus, consistent with our previous work [21,22] attention switching did not limit the overall success of encoding items into memory, but rather reduced the selectivity of encoding task-relevant vs. irrelevant material.

#### Memory selectivity

**Fig 3:**
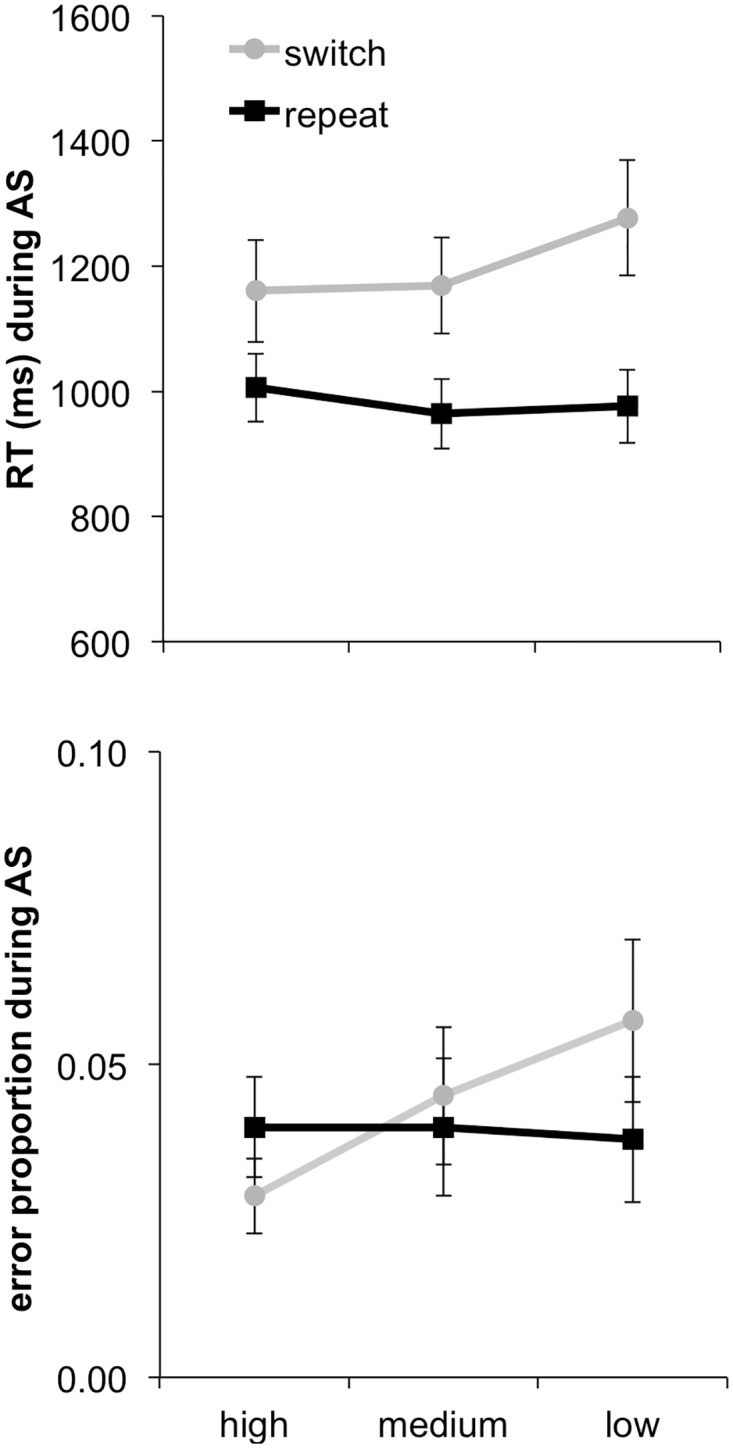
RTs and error rates for high, medium (med), and low memory selectivity trials separately for switch and repeat trials. AS = Attention switching. Error bars indicate standard errors of the mean.

The preceding results indicate that attention switching reduced the selectivity of memory. We have previously shown in a similar paradigm that trial-by-trial variation in memory selectivity indexes the effectiveness of cognitive control during earlier attention switching, as reflected in RTs and error rates [21]. Replicating this analysis here, we analysed differences in RTs and error rates in the earlier attention-switching phase after dividing trials into three bins according to the memory selectivity score derived from the later memory ratings of items from a given trial (Fig 3).

These data were entered into two ANOVAs separately for RTs and error rates, with variables Memory Selectivity (high, medium and low) and Switch. These analyses identified the already-described main effect of Switch, *F*(1, 15) = 30.60, *p* < .01, in the RT data. The main effect of Memory Selectivity was marginally significant, *F*(2, 30) = 2.93, *ε* = .703, *p* = .083, and the interaction between Switch and Memory Selectivity reached significance in the RTs, *F*(1, 15) = 5.11, *p* < .05. Separate one-way ANOVAs were conducted for switch and repeat trials to follow-up on these effects. The ANOVA on the repeat-trial data revealed no significant effect of Memory Selectivity, *F*(2, 30) = 1.47, *p* = .247. The ANOVA on the switch-trial data, however, indicated an effect of Memory Selectivity on the RTs, *F*(2, 30) = 4.84, *p* < .05. A significant linear trend, *F*(1, 15) = 6.03, *p* < .05, revealed that RTs increased overall with decreasing memory selectivity. Thus, our results are consistent with findings from our prior studies [21] that switch trials are highly reliant on attentional processes, whereas repeat trials are less susceptible to fluctuations in attention. Corresponding numerical trends were apparent in an analysis of the error rate data, but no effects reached statistical significance, again potentially due to low error rates overall.

Thus, switch trials appeared to be more susceptible to the effectiveness with which attention was allocated on a given trial, as evident in the increasing RTs with decreasing memory selectivity. Accordingly, in a paradigm in which subjects switched their attention between stimuli, but did not change the required classification rule, we replicated the pattern observed in our prior work that combined attention and categorization switching.

#### Global memory

The focus of the current paper was to contrast the effects of differences in selective vs. global memory encoding. Thus, our next analysis assessed differences in earlier performance associated with later differences in global memory (i.e., memory of attended and unattended information overall, regardless of task-relevance). The global memory measure was calculated in parallel to the memory selectivity measure, in that trials were divided into three bins based on the memory ratings that participants gave to the attended and unattended item of each bivalent trial, but now summing rather than subtracting these ratings.

**Fig 4:**
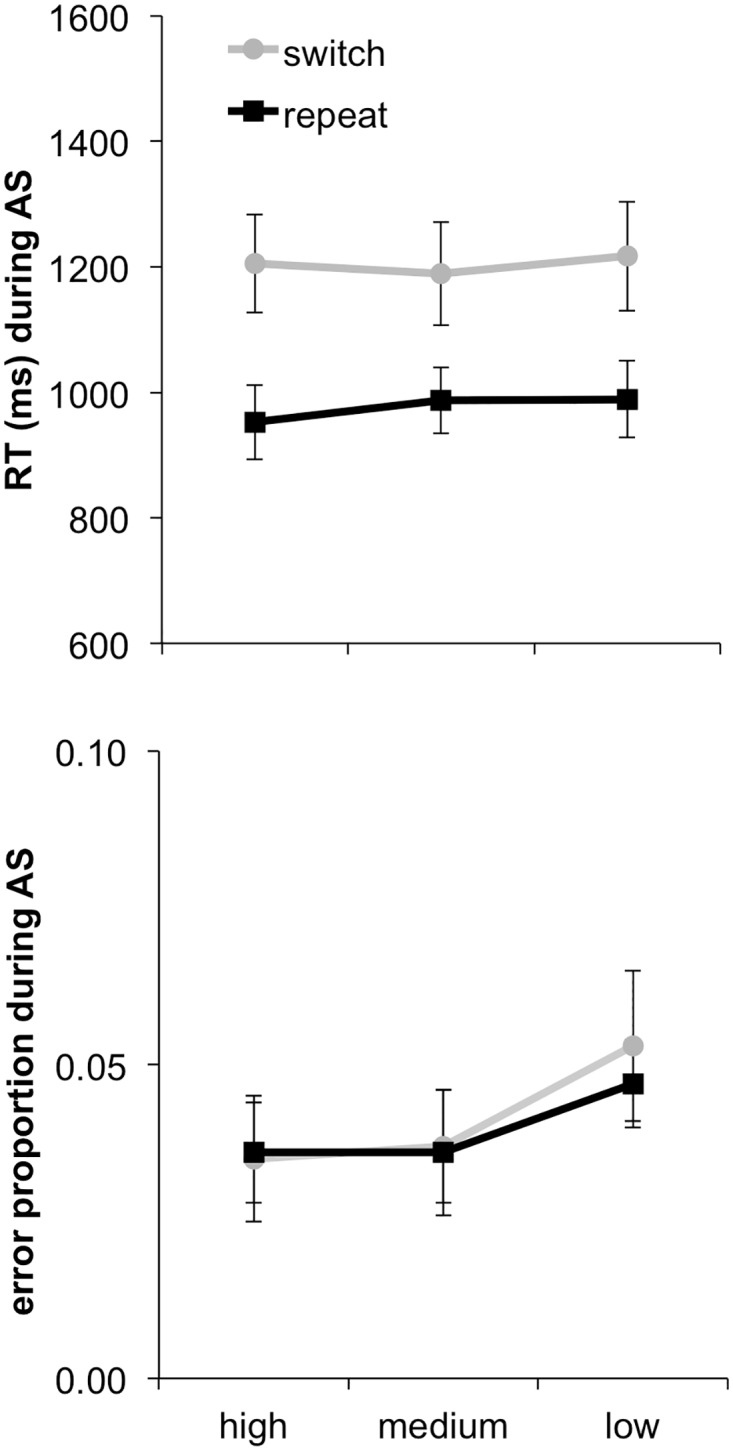
RTs and error rates for high, medium (med), and low global memory trials separately for switch and repeat trials. AS = Attention switching. Error bars indicate standard errors of the mean.

Analysis of the global memory data (Fig 4) revealed only a main effect of Switch in the RT data, *F*(1, 15) = 32.43, *p* < .01 (i.e., the switch cost), but again not in the error rates, *F* < 1. There was no significant main effect of Global Memory in the RT analysis, *F* < 1, nor for error rates, *F*(2, 30) = 1.16, *p* = .329, and no reliable interaction between Global Memory and Switching for RTs, *F*(2, 30) = 1.10, *p* = .345, or error rates, *F* < 1. Thus, there was no clear relationship between the overall amount of information successfully encoded and the level of task performance when initially encountering this information.

#### Relationship between memory selectivity and global memory

In principle, it is possible that Memory Selectivity and Global Memory could be highly correlated. For example, if memory for unattended items was uniformly poor and meaningful variation in recognition memory was only observed for attended items, the two measures would effectively measure the same construct. In reality, however, substantial variability was observed in memory ratings for both attended and unattended items, such that the full range of possible Memory Selectivity and Global Memory scores was consistently observed. Crucially, the two measures were only very weakly related to each other: The overall mean of Fisher *z*-transformed within-subject correlations was low and not significant, *r = -*.040, *t*_*15*_ *=* 1.46, *p =* .165 (similarly when analysed separately for repeat trials, *r = -*.035, *t*_*15*_ < 1, and switch trials, *r = -*.041, *t*_*15*_ *=* 1.86, *p =* .083). These analyses support our intention and interpretation that the two variables measure different constructs.

### ERP results

Collectively, our behavioural results replicate and extend previous findings [21,31]. Recognition memory scores for seen objects and words were predictive of earlier attention switching performance, but only when considered in terms of memory selectivity and not global memory scores. With these behavioural results established, we next assessed the ERP correlates of long-term memory encoding in analyses of the EEG data.

We report analyses focussing on both cue-locked and stimulus-locked activity from the attention-switching phase. Of primary interest here was whether subsequent memory effects would be evident as effects of memory selectivity (indicating that subsequent memory effects reflect enhanced processing of task relevant material), effects of global memory (suggestive of more general processes), or both. Regarding correlates of switching, we were primarily interested in the question of whether ERPs associated with successful remembering are similar to those of successful task performance.

### Cue-related potentials

#### Memory selectivity

The first goal of this analysis was to investigate whether prestimulus subsequent memory effects can be detected using the measure of memory selectivity (difference in memory ratings between the attended and unattended items)—such anticipatory effects would indicate preparation for selective processing. To investigate ERP effects associated with memory selectivity and its interactions with other variables, the ERP data were entered in an ANOVA with variables Switch (switch/repeat), Time (0–300, 300–600, 600–900, 900–1200 ms after cue onset), Location (frontal/posterior), Material (object/word), and Memory Selectivity (high/low).

**Fig 5:**
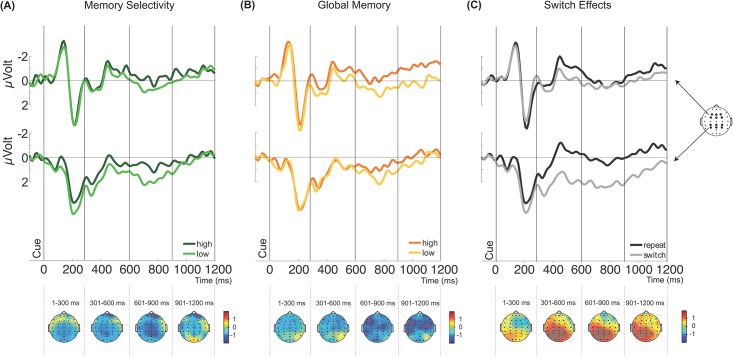
Cue-locked potentials for Memory Selectivity, Global Memory, and Switching, measured at the frontal electrodes F3, FZ, F4, FC3, FCZ, and FC4 (upper) and posterior electrodes CP3, CPZ, CP4, P3, PZ, and P4 (lower), as well as topographic maps. (A) Top: ERP waveforms for high and low memory selectivity trials, both time-locked to the onset of the cue. Bottom: The scalp topography of the average signal differences between high and low memory selectivity trials in steps of 300 ms starting with cue-onset. (B) Top: ERP waveforms for high and low global memory trials, both time-locked to the onset of the cue. Bottom: The scalp topography of the average signal differences between high and low global memory trials in steps of 300 ms starting with cue-onset. (C) Top: ERP wave forms for switch and repeat trials, both time-locked to the onset of the cue. Bottom: The scalp topography of the average signal differences between switch and repeat trials in steps of 300 ms starting with cue-onset. Plots show data collapsed across material and switching (A and B), and material (C).

Cue-locked ERP waveforms for the frontal and posterior electrode clusters, as well as scalp topographies, are shown in Fig 5A, where effects are collapsed across the variables Material and Switch. High memory selectivity trials were characterised by more negative scalp voltages over posterior midline sites than trials with low memory selectivity, peaking 600–900 ms after cue onset. However, no significant main effect of Memory Selectivity, *F*(1, 13) = 2.16, *p* = .166, with no significant interactions, for example, between Memory Selectivity and Switch, *F*(1, 13) = 1.60, *p* = .228, Memory Selectivity and Time, *F*(1, 13) = 1.03, *p* = .390 or Memory Selectivity and Location, *F* < 1, were found. The main effect of switching was only marginally significant, but descriptively displayed the expected topography (trend for more positive switch than repeat trials, [37–39], *F*(1, 13) = 4.00, *p* = .067). Thus, analysis of cue-locked Memory Selectivity data did not reveal robust evidence for selective preparation processes.

#### Global memory

A corresponding analysis focussed on the effects of global memory. The same conditions as specified above were used, with the Memory Selectivity variable replaced with the variable of Global Memory. High minus low global memory effects were descriptively also characterized by negative topographies, here with maxima in left and right central to fronto-central areas (Fig 5B; effects again collapsed across variables Material and Switch). ERP differences associated with global memory, and its interactions with other variables, were also assessed for the cue ERPs: An ANOVA on the Global Memory data (including variables Switch, Time, Location, Material, and Global Memory), revealed no significant main effect of Global Memory, *F*(1, 13) = 1.44, *p* = .251, and no significant interactions between Global Memory and other variables (all *F*s < 2.74, *p*s > .05). Thus, analysis of cue-locked global memory effects did not find evidence for general effects of preparation.

#### Switch-related effects

The second goal of the analysis of the cue ERP data was to investigate ERP effects of switching, to contrast them with the memory-related effects. In contrast to the weak modulation of preparatory potentials as a function of Memory Selectivity and Global Memory, we robustly replicated the finding of a posterior switch-related positivity (Fig 5C, see [38,39,48]). Thus, an ANOVA with variables Switch (switch/repeat), Time (0–300, 300–600, 600–900, and 900–1200 ms after cue onset), Location (frontal/posterior), and Material (object/word), revealed a significant main effect of Switch, *F*(1, 13) = 5.55, *p* < .05, indicating overall more positive ERPs for switch versus repeat trials. In addition, this analysis revealed a marginally significant interaction between Switch and Time, *F*(3, 39) = 3.21, ε = .447, *p* = .081, and a significant interaction between Switch and Location, *F*(1, 13) = 14.80, *p* < .01. These interactions were further qualified by a three-way interaction between Location, Switch, and Time, *F*(3, 39) = 2.95, *p* < .05. Switch compared to repeat trials were more positive over posterior sites, an effect peaking in the 300–600 ms and 600–900 ms time windows (switch-related posterior positivity, [see e.g., [38]]), but significant in each 300 ms time window from 300–1200 ms post-cue, *t*s < -3.18, *p*s < .01. Thus, we observed robust switch-related effects in the preparation phase that mirrored previously-observed ERP correlations of preparation in task switching.

Although it might initially appear as if the cue-locked switch effect and memory selectivity effect show opposing topographies (posterior positivity for the switch effect vs. negativity for memory selectivity), the topographies in fact converge in showing greater negativity associated with higher memory selectivity: Switch trials usually result in lower selectivity than repeat trials, such that the switch effect effectively plots lower selectivity (switch) minus higher selectivity (repeat) trials. This is the inverse of the Memory Selectivity effect (which displays high minus low memory selectivity trials), accounting for the opposite polarity of the effects. Thus, effects of memory selectivity (though weak) and switching resembled each other in the cue ERPs in topography and timing.

Together, the results of the preceding analyses suggest that the cue ERPs display the well-documented effects of switching. In contrast, subsequent memory effects assessed with the measures of memory selectivity and global memory were not reliable in this phase. Nevertheless, ERP differences associated with differences in memory selectivity, though weak, showed a descriptively similar pattern to switch effects with regards to topography and timing.

### Stimulus-related potentials

The stimulus ERPs were analysed similarly to the cue ERPs, comparing successive 300 ms time windows (0–300, 300–600, 600–900 and 900–1200 ms after stimulus onset), separately for switch and repeat trials, and as a function of Location (frontal/posterior) and Material (object/word).

#### Memory selectivity

**Fig 6:**
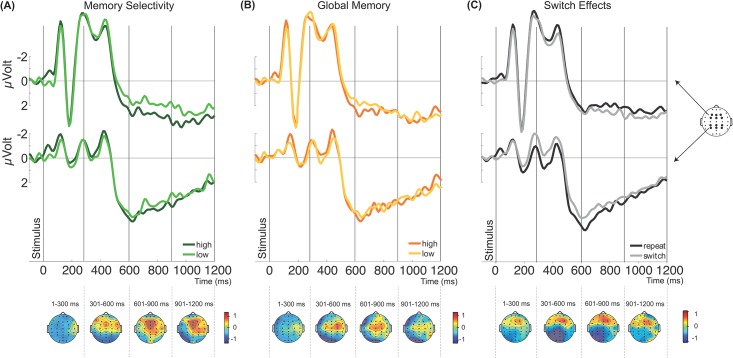
Stimulus-locked potentials for Memory Selectivity, Global Memory, and Switching, measured at the frontal electrodes F3, FZ, F4, FC3, FCZ, and FC4 (upper) and posterior electrodes CP3, CPZ, CP4, P3, PZ, and P4 (lower), as well as topographic maps. (A) Top: ERP waveforms for high and low memory selectivity trials, both time-locked to the onset of the stimulus. Bottom: The scalp topography of the average signal differences between high and low memory selectivity trials in steps of 300 ms starting with stimulus-onset. (B) Top: ERP waveforms for high and low global memory trials, both time-locked to the onset of the stimulus. Bottom: The scalp topography of the average signal differences between high and low global memory trials in steps of 300 ms starting with stimulus-onset. (C) Top: ERP wave forms for switch and repeat trials, both time-locked to the onset of the stimulus. Bottom: The scalp topography of the average signal differences between switch and repeat trials in steps of 300 ms starting with stimulus-onset. Figures show data collapsed across material and switching (A and B), and material (C).

A first ANOVA investigated whether memory selectivity effects resembled the well-documented stimulus-locked subsequent memory effects, which are often observed as frontal or fronto-central positive slow waves (e.g., [12,15,16]). ERP plots and topographies for this analysis are given in Fig 6A.

Any subsequent memory effect relating to processing selectivity should be apparent as a sustained positivity over frontal electrodes on trials with high memory selectivity. Precisely this pattern was observed (Fig 6A), as reflected in a reliable interaction between Time and Memory Selectivity, *F*(3, 39) = 4.26, ε = .651, *p* < .05. Scalp voltage was reliably more positive on trials with high memory selectivity, reliably so in the final analysis time window 900–1200 ms poststimulus, *t*(13) = 2.31, *p* < .05, with the same pattern in the immediately preceding window, though not reliable, *t*(13) = 1.70, *p* = .113. These subsequent memory effects were numerically much larger over frontal electrode locations (0.871 *μV* vs. 0.218 *μV*), although the interaction between Time, Memory Selectivity and Location did not reach significance, *F*(3, 39) = 2.35, ε = .153, *p* = .115. Thus, the effects of Memory Selectivity resembled previously described frontal slow-wave subsequent memory effects, both in topography and timing [12,14,18].

#### Global memory

Similar to the memory selectivity effect, global memory effects (high minus low global memory trials) also showed a positive topography, but peaking at more central electrodes (see Fig 6B). An ANOVA on the global memory data with variables Switch, Time, Location, Material, and Global Memory revealed no main effect of Global Memory, *F* < 1, and no significant interactions between Global Memory and Time, *F* < 1, Global Memory and Switch, *F* < 1, or Global Memory and Location, *F*(1, 13) = 1.46, *p* = .248. Only a significant interaction between Location, Material, and Global Memory was found, *F*(1, 13) = 5.31, *p* < .05. However, separate ANOVAs for high and low global memory trials did not reveal any significant interaction effects (all *p* > .1), and the overall effect appeared to reflect idiosyncratic differences across material types rather than any consistent effect of global memory.

Visual inspection of the topographic plots indicated that effects of Global Memory were largest at central electrodes (C3, Cz, C4), but our primary analysis of frontal and posterior clusters excluded this electrode cluster. To ensure that the comparison of memory selectivity and global memory effects was not biased by the way the electrodes were divided for analysis, the ERP data of these vertex electrodes was entered into an additional, simplified ANOVA. This ANOVA with variables Global Memory and Time only revealed a significant effect of Time, *F*(3, 39) = 15.49, ε = .485, *p* < .01, but no main effect of Global Memory, *F* < 1, nor any reliable interaction between Time and Global Memory *F*(3, 39) = 1.31, *ε* = .652, *p* = .286. Thus, altogether the stimulus ERPs showed little evidence for systematic subsequent memory effects associated with the total amount (global memory) of information encoded regardless of task relevance.

#### Switch-related effects

Stimulus-locked ERPs for switch and repeat trials are shown in Fig 6C. The topographies for switch minus repeat trials were characterized by a pattern of posterior negativities and frontal positivities, most pronounced between 300 and 900 ms. (Thus, in contrast to the cue ERPs, memory selectivity and switch effects did *not* follow a similar pattern in the stimulus locked data). An ANOVA with factors of Switch, Material type, Location, and Time revealed main effects of Location, *F*(1, 13) = 6.95, *p* < .05, with a more positive voltage at posterior sites, and of Time, *F*(3, 39) = 13.04, ε = .488, *p* < .01, with a significant linear trend indicating that the ERP increased in positivity over time, *F*(1, 13) = 13.77, *p* < .01. These main effects were qualified by several interactions.

The interaction between Switch and Location was significant, *F*(1, 13) = 5.18, *p* < .05, and indicated that scalp voltage was more positive over frontal electrodes on switch trials than repeat trials, whereas scalp voltage was more negative over posterior sites for the same contrast. Pairwise contrasts however revealed that switch and repeat trials did not differ significantly at frontal, *t*(13) = -1.14, *p =* .277, or posterior, *t <* 1, sites. A significant difference was only observed for repeat trials between frontal and posterior electrodes, *t*(13) = -3.01, *p* < .05, and a marginally reliable difference for switch trials between frontal and posterior electrodes, *t*(13) = -2.03, *p* = .063. These effects of switching might represent residual activity from the prestimulus phase, rather than independent stimulus-locked effects [49].

Moreover, the interaction between Time and Location reached significance, *F*(3, 39) = 9.83, ε = .560, *p* < .01, as did the three-way interaction between Time, Location, and Material, *F*(3, 39) = 3.08, *p* < .05. Follow-up analysis revealed a significant interaction between Time and Material at posterior electrodes, *F*(3, 39) = 3.68, *p* < .05, but not at frontal electrodes, *F*(3, 39) = 2.32, ε = .541, *p* = .131. Scalp voltage was more positive on object trials than word trials in the 300–600 ms time window at posterior electrodes, *t*(13) = -2.63, *p* < .05, with no significant effects of material type apparent in other time windows (all other *t <* 1.76, *ps* > .10).

Thus, the results of the analyses of switch related effects suggest that the stimulus ERPs display less consistent effects of switching than the cue ERPs. The topographical pattern of the stimulus-locked effect was nevertheless consistent with previous studies (e.g., [38,40]).

To summarise the above results, in the preparation phase we found limited evidence for subsequent memory effects, for measures of either memory selectivity or global memory. In contrast, robust switch effects were observed during this preparation period. In this phase, memory selectivity effects, though weak, showed a similar pattern to switch effects with regards to topography and timing. Critically, during the post-stimulus phase, we found evidence for subsequent memory effects that were unique to the measure of memory selectivity, and were not clearly observed in measures of global memory. In this phase, switch-related effects were weaker and diverged in timing and topography from the subsequent memory effects.

## Discussion

The present study aimed to characterise and contrast the ERP correlates of successful memory encoding and successful attention switching. Specifically, two measures of memory success—memory selectivity and global memory—were used to assess whether previously observed ERP subsequent memory effects reflect focused encoding of task-relevant information (memory selectivity), general encoding success (global memory), or both. We additionally explored how ERP correlates of successful encoding relate to control processes observed in attention switching.

### Memory selectivity and global memory

A critical feature of the present study was the inclusion of separate task-relevant and irrelevant items on each encoding trial, enabling us to dissociate *memory selectivity*—the difference in recognition memory confidence for task-relevant vs. irrelevant items—from what we term *global memory*—that is, recognition memory confidence averaged across both relevant and irrelevant items. Our behavioural results replicated previous findings [21,22]: The cognitive demands of attention switching primarily impacted on later memory in terms of its selectivity for task-relevant information, and encoding task performance scaled only with later memory selectivity, but not global memory. Indeed, memory for task-irrelevant items was, if anything, improved rather than impaired on trials requiring an attention switch.

This observation of improved memory for task-irrelevant items on switch trials helps to address an important concern with our measure of global memory, which relates to the possibility that unattended items might not be encoded, or might even be actively suppressed, because of the interference they could potentially cause. If so, our measure of global memory might underestimate the amount of information that could in principle be encoded, such that global memory would be better estimated by assessing memory for items that were not ‘actively’ unattended (e.g., a third class of items other than objects and words that were never task-relevant, or were incidental associates of the target item). However, we find better memory for unattended items appearing on switch trials, where active suppression is presumed to be strongest (cf. [50]). Meanwhile, recent evidence indicates that task-irrelevant items that were recently attended during task switching receive more attention [51] and are better remembered [52] than items that are never task-relevant and attended. Collectively, this evidence indicates that task-irrelevant features of bivalent stimuli are both processed and encoded, if anything more strongly than other incidentally present information. On this basis, we are confident that our global memory measure provides a sensitive index of the degree to which information is encoded regardless of task relevance.

Altogether, our behavioural findings replicate what we have observed previously, notably using for the first time a paradigm in which the same semantic judgement was required on every trial. Our previous studies had confounded switches of attention (from object to word or vice versa) with switches in the semantic judgement required (from natural/human-made to abstract/concrete or vice versa). Evidently, a simple switch of attention between objects and words is sufficient to impact strongly on successful encoding—further evidence of the close interrelationship between attention and memory [20]. Of interest, then, given these behavioural results, are the corresponding ERP markers of subsequent memory seen during the encoding phase, separately for our measures of selective vs. global memory.

We observed robust subsequent memory effects only in analyses of stimulus ERPs, but not in our exploratory analyses of pre-stimulus activity in the cue ERPs. Previous studies have shown that stimulus-locked subsequent memory effects typically exhibit a fronto-central or centro-parietal positive topography [5,14,15,53,54]. Crucially, although subsequent memory differences were evident in our ERP waveforms as a function of both memory selectivity and global memory, careful inspection made clear that only memory selectivity effects were robust and fit the profile of previously-observed subsequent memory effects.

ERP differences as a function of memory selectivity emerged around 500 ms post-stimulus, and were most robustly observed in the final 900–1200 ms window of analysis. Moreover, the effects were focused over frontal midline sites, as one would expect of subsequent memory effects in a task requiring elaborative encoding such as the natural vs. human-made task used here [13,15]. In contrast, ERP differences as a function of global memory peaked around 500 to 700 ms post-stimulus, were short-lived, and were not statistically reliable even when considering only electrodes around the vertex where the differences were maximal. The observed pattern was, however, similar to centro-parietal subsequent memory effects sometimes observed [5,11,15]. These effects, which are often less reliable than the frontal subsequent memory effects [17], have been related to rote or perceptual encoding processes. These may be factors that are influencing global memory as well.

Elsewhere [20] we have identified factors that affect behavioral measures of memory selectivity, by manipulating control demands. Future research will need to assess whether the same variables that affect memory selectivity behaviorally also influence its neural correlates, and subsequent memory effects. A more open question concerns which factors, if any, might affect global memory. Likely candidates for manipulations that would affect global memory are distraction from the task, interference, or memory load.

On the basis of previous literature (cf. [7,27,28]), we also explored prestimulus subsequent memory effects in analyses of the cue ERP data. The exact nature of the expected topography was difficult to predict because prestimulus effects have proven to be less reliable than stimulus-locked subsequent memory effects and have sometimes been shown to vary with the task or stimuli employed [26,28] and sometimes not (e.g., [27]). The results of this exploratory cue-locked analysis were inconclusive, with no consistent evidence of subsequent memory effects in terms of either memory selectivity or global memory: The numerical trends were towards an enhanced slow-wave negativity with broad topography on trials with both high memory selectivity and high global memory, and with some differences in the precise timecourse and topography of these effects. However, the lack of statistically reliable differences argues against drawing strong conclusions beyond the point that cue- and stimulus-locked subsequent memory effects are dissociable from each other, in that they do not necessarily co-occur [7,27]—here we observed robust poststimulus differences as a function of memory selectivity, but no clear corresponding prestimulus effects. One potential limitation of the current study that might be of relevance in this context is the restricted number of encoding trials that were available in the different conditions, due to the necessity of a long retrieval phase. Future studies will likely be necessary to further explore ERP prestimulus subsequent memory effects.

In summary, our analyses suggest that effects of memory selectivity and global memory are dissociable. Moreover, taken with the finding that global memory did not scale with encoding task performance, the ERP results are consistent with the notion that frontal subsequent memory effects reflect selective processing in elaborative encoding tasks: If a measure of global memory is used, no effects on task performance are observed, and ERP-subsequent memory effects are not reliable and descriptively more posterior-focused. Overall, therefore, our findings suggest that frontal subsequent memory effects associated with elaborative encoding reflect the establishment of a focused cognitive set for processing of task-relevant information.

### Switching and memory selectivity

The second key goal of this study was to contrast ERP correlates of attention switching with subsequent memory effects. Behaviourally, we observed significant modulation of later memory by attention switching, specifically as a reduction in selectivity of memory [10,21,22]. Replicating earlier findings, the magnitude of this memory selectivity effect was predictive of earlier attention switching performance, with greater selectivity associated with faster switch-trial RTs. Given this strong behavioural association between attention switching and later memory, we might expect to observe common ERP correlates of successful encoding and switching. However, the ERP data were more notable for differences in these ERP correlates rather than for similarities.

Considering first the cue ERPs, robust ERP differences were apparent in the comparison between prestimulus potentials on switch and repeat trials, which contrast with the weak and non-significant effects observed in our subsequent memory effect analyses. The switching-related differences were maximal over posterior sites, with some indication of left-lateralization, consistent with previous findings that have been interpreted as increased preparation processes in switch trials (e.g., [37,38]). As in previous task and attention switching studies using long preparation intervals, switch effects descriptively started to diminish towards the end of the preparation interval [38], perhaps indicating that preparation processes were approaching completion.

However, although robust preparatory potentials were only apparent in relation to attention switching not subsequent memory, a qualitatively similar pattern could be noted in prestimulus memory selectivity effects. Specifically, a sustained positive wave over posterior scalp sites was apparent (at least as a numerical trend) on trials with high memory selectivity, similar to that observed on repeat trials—i.e., exactly those trials that, behaviourally, were associated with higher levels of memory selectivity. Taken with evidence that factors known to improve preparation in attention switching produce corresponding improvements in memory selectivity [22], it seems premature to conclude that preparatory processes associated with switching and subsequent memory are fundamentally different—it remains possible that they differ in degree rather than in kind.

In the stimulus-locked ERP data, in contrast, we observed clear and qualitative differences between attention switching and subsequent memory. As described above, comparison of poststimulus ERPs on trials with high vs. low memory selectivity revealed a sustained positive wave that was maximal over frontal sites and most consistently observed in the final analysis window (900–1200 ms). By contrast, poststimulus differences between switch and repeat trials were apparent in earlier time windows as relatively greater positive voltage over frontal electrodes and relatively greater negative voltage over posterior electrodes on switch trials than repeat trials. Crucially, there was no hint that repeat trials (which were marked by higher memory selectivity scores) were associated with the same sustained frontal positivity as identified in the direct contrast between trials with high vs. low memory selectivity. Indeed, if anything, the numerical trend was for greater positive voltages on switch trials in late time-windows.

This divergence indicates that processes associated with attention switching and memory encoding differ in the stimulus phase, despite the interactions between attention and memory observed behaviourally. Put together, the behavioural and ERP findings suggest that effectively preparing an encoding task (or adapting a ‘task set’) may be necessary but not sufficient to guarantee successful encoding in memory. Following this interpretation, prestimulus subsequent memory effects may be more related to differences in a neural state or ‘task set’ (cf. [7]), while post-stimulus effects may be more related to the actual processing that is applied to the stimulus. While adopting an adequate neural state might itself lead to more successful stimulus-processing, stimulus-encoding itself naturally has to wait until the stimulus is shown, leading to more prominent subsequent memory effects in the stimulus phase. Variability in the effectiveness of stimulus processing—which might reflect, amongst other things, fluctuations in task focus across time, idiosyncratic differences in participants’ treatment of individual stimulus items, or stimulus-driven effects—might then impact on subsequent memory (and its ERP correlates) regardless of the initial success with which encoding was prepared. The same might not be true for switching, which appears to benefit more from stimulus-independent preparation processes. In line with this argument, it has been observed that poststimulus EEG switching effects, such as the posterior negativity also observed in the current study, may be reduced with increasing preparation [40]. This observation suggests that preparation can reduce the need for switch-specific control processes in the stimulus-phase, in contrast to stimulus-encoding.

Overall, the ERP results indicate that there is a complex and nuanced relationship between memory and cognitive control. A strong interpretation of the behavioural data collected here and elsewhere [21,22] could be that memory is nothing more than enduring traces of processing that is guided by top-down cognitive control (cf. [31]). This interpretation provides an attractively simple account of the behavioural interactions between memory and cognitive control in attention switching. The current ERP data indicate, however, that this interpretation may be too simple, with evidence of substantial variability in encoding success that is independent of top-down control but that is reflected in well-established neural markers of subsequent memory.

### Conclusion

Collectively, the present findings shed new light on the role of top-down control in successful memory encoding. Previous studies have documented robust ERP subsequent memory effects, but leave open whether these effects relate to selective encoding of task-relevant information, general encoding success, or both. Our results provide clear evidence in favour of the first hypothesis, and as such suggest that subsequent memory effects in elaborative encoding tasks are at least partly the result of attentional focussing and effective top-down control. In this regard, our findings are consistent with current theories that emphasize the role of selective attention in memory [31,55]. However, differences in the ERP correlates of attention switching and successful encoding, particularly during the poststimulus period, indicate important limitations in the scope of this conclusion: Substantial variability in encoding success, as reflected in frontal subsequent memory effects, occurs independent from prestimulus demands for top-down cognitive control.

## Supporting Information

S1 File Data. (XLSX)

## References

[1] Craik FIM, Govoni R, Naveh-Benjamin M, Anderson ND. The effects of divided attention on encoding and retrieval processes in human memory. Journal of Experimental Psychology-General 1996;125(2):159–80. 8683192 10.1037//0096-3445.125.2.159

[2] Bower GH, Clark MC, Lesgold AM, Winzenz D. Hierarchical retrieval schemes in recall of categorized word lists. Journal of Verbal Learning and Verbal Behavior 1969;8:323–43.

[3] Craik FI, Tulving E. Depth of processing and the retention of words in episodic memory. Journal of Experimental Psychology: General 1975;104(3):268.

[4] Morris CD, Bransford JD, Franks JJ. Levels of processing versus transfer appropriate processing. Journal of Verbal Learning and Verbal Behavior 1977;16(5):519–33.

[5] Sanquist TF, Rohrbaugh JW, Syndulko K, Lindsley DB. Electrocortical signs of levels of processing—perceptual analysis and recognition memory. Psychophysiology 1980;17(6):568–76. 7443924 10.1111/j.1469-8986.1980.tb02299.x

[6] Galli G, Gebert AD, Otten LJ. Available processing resources influence encoding-related brain activity before an event. Cortex 2013;49(8):2239–48. 10.1016/j.cortex.2012.10.011 23219383 PMC3764337

[7] Otten LJ, Quayle AH, Akram S, Ditewig TA, Rugg MD. Brain activity before an event predicts later recollection. Nat Neurosci 2006;9(4):489–91. 10.1038/nn1663 16501566

[8] Monsell S. Control of mental processes In: Bruce V, editors. Unsolved mysteries of the mind. Hove, E. Sussex: Erlbaum; 1996h p. 93–148.

[9] Sakai K. Task set and prefrontal cortex. Annual Review of Neuroscience 2008;31:219–45. 10.1146/annurev.neuro.31.060407.125642 18558854

[10] Krebs RM, Boehler CN, De Belder M, Egner T. Neural conflict-control mechanisms improve memory for target stimuli. Cereb Cortex 2015;25(3):833–43. 10.1093/cercor/bht283 24108799 PMC4400531

[11] Paller KA, Kutas M, Mayes AR. Neural correlates of encoding in an incidental-learning paradigm. Electroencephalogr Clin Neurophysiol 1987;67(4):360–71. 2441971 10.1016/0013-4694(87)90124-6

[12] Karis D, Fabiani M, Donchin E. "P300" and memory—individual-differences in the von restorff effect. Cogn Psychol 1984;16(2):177–216.

[13] Guo C, Zhu Y, Ding J, Fan S, Paller KA. An electrophysiological investigation of memory encoding, depth of processing, and word frequency in humans. Neurosci Lett 2004;356(2):79–82. 14746868 10.1016/j.neulet.2003.09.049

[14] Fernandez G, Weyerts H, Tendolkar I, Smid HGOM, Scholz M, Heinze HJ. Event-related potentials of verbal encoding into episodic memory: Dissociation between the effects of subsequent memory performance and distinctiveness. Psychophysiology 1998;35(6):709–20. 9844432

[15] Fabiani M, Karis D, Donchin E. Effects of mnemonic strategy manipulation in a von restorff paradigm. Electroencephalogr Clin Neurophysiol 1990;75(2):22–35. 1688770 10.1016/0013-4694(90)90149-e

[16] Schott B, Richardson-Klavehn A, Heinze HJ, Düzel E. Perceptual priming versus explicit memory: Dissociable neural correlates at encoding. J Cogn Neurosci 2002;14(4):578–92. 10.1162/08989290260045828 12126499

[17] Fabiani M, Donchin E. Encoding processes and memory organization—a model of the vonrestorff effect. J Exp Psychol Learn Mem Cogn 1995;21(1):224–40. 7876771 10.1037//0278-7393.21.1.224

[18] Paller KA, Wagner AD. Observing the transformation of experience into memory. Trends Cogn Sci 2002;6(2):93–102. 15866193 10.1016/s1364-6613(00)01845-3

[19] Rugg MD. ERP studies of memory In: Coles MGH, editors. Electrophysiology of mind: Event-related brain potentials and cognition. Oxford: Oxford University Press; 1995m p. 132–70.

[20] Chun MM, Turk-Browne NB. Interactions between attention and memory. Curr Opin Neurobiol 2007;17(2):177–84. 10.1016/j.conb.2007.03.005 17379501

[21] Richter FR, Yeung N. Memory and cognitive control in task switching. Psychol Sci 2012;23(10):1256–63. 10.1177/0956797612444613 22972906

[22] Richter, F. R. (2013). The control of task sets and long-term memory (Doctoral dissertation, University of Oxford).

[23] Richter FR, Yeung N. Neuroimaging studies of task switching In: Task switching and cognitive control.; 2014j p. 237–71.

[24] Kim C, Johnson NF, Cilles SE, Gold BT. Common and distinct mechanisms of cognitive flexibility in prefrontal cortex. Journal of Neuroscience 2011;31(13):4771–9. 10.1523/JNEUROSCI.5923-10.2011 21451015 PMC3086290

[25] Chiu YC, Egner T. Inhibition-Induced forgetting results from resource competition between response inhibition and memory encoding processes. J Neurosci 2015;35(34):11936–45. 10.1523/JNEUROSCI.0519-15.2015 26311775 PMC4549404

[26] Galli G, Choy TL, Otten LJ. Prestimulus brain activity predicts primacy in list learning. Cogn Neurosci 2012;3(3–4):160–7. 10.1080/17588928.2012.670105 22888370 PMC3413908

[27] Otten LJ, Quayle AH, Puvaneswaran B. Prestimulus subsequent memory effects for auditory and visual events. J Cogn Neurosci 2010;22(6):1212–23. 10.1162/jocn.2009.21298 19583467 PMC3881065

[28] Padovani T, Koenig T, Brandeis D, Perrig WJ. Different brain activities predict retrieval success during emotional and semantic encoding. J Cogn Neurosci 2011;23(12):4008–21. 10.1162/jocn_a_00096 21812556

[29] Evans LH, Herron JE, Wilding EL. Direct real-time neural evidence for task-set inertia. Psychol Sci 2015. 10.1177/0956797614561799 PMC4361352 25626443

[30] Yeung N, Nystrom LE, Aronson JA, Cohen JD. Between-task competition and cognitive control in task switching. Journal of Neuroscience 2006;26(5):1429–38. 10.1523/JNEUROSCI.3109-05.2006 16452666 PMC6675498

[31] Richter FR, Yeung N. Corresponding influences of top-down control on task switching and long-term memory. Q J Exp Psychol (Hove) 2015;68(6):1124–47. 25337969 10.1080/17470218.2014.976579

[32] Evans LH, Williams AN, Wilding EL. Electrophysiological evidence for retrieval mode immediately after a task switch. Neuroimage 2015. 10.1016/j.neuroimage.2014.12.068 PMC4334665 25562822

[33] Padovani T, Koenig T, Eckstein D, Perrig WJ. Sustained and transient attentional processes modulate neural predictors of memory encoding in consecutive time periods. Brain Behav 2013;3(4):464–75. 10.1002/brb3.150 24381815 PMC3869685

[34] Karayanidis F, Jamadar SD. Event-Related potentials reveal multiple components of proactive and reactive control in task switching In: Task Switching and Cognitive Control. Oxford University Press; 2014m p. 200.

[35] Otten LJ, Rugg MD. When more means less: Neural activity related to unsuccessful memory encoding. Current Biology 2001;11(19):1528–30. 11591321 10.1016/s0960-9822(01)00454-7

[36] Falkenstein M, Hoormann J, Hohnsbein J, Kleinsorge T. Short-term mobilization of processing resources is revealed in the event-related potential. Psychophysiology 2003;40(6):914–23. 14986844 10.1111/1469-8986.00109

[37] Poljac E, Yeung N. Dissociable neural correlates of intention and action preparation in voluntary task switching. Cerebral Cortex 2014. 10.1093/cercor/bhs326 PMC3888369 23104682

[38] Karayanidis F, Coltheart M, Michie PT, Murphy K. Electrophysiological correlates of anticipatory and poststimulus components of task switching. Psychophysiology 2003;40(3):329–48. 12946108 10.1111/1469-8986.00037

[39] Karayanidis F, Provost A, Brown S, Paton B, Heathcote A. Switch-specific and general preparation map onto different ERP components in a task-switching paradigm. Psychophysiology 2011;48(4):559–68. 10.1111/j.1469-8986.2010.01115.x 20718932

[40] Kieffaber PD, Hetrick WP. Event-related potential correlates of task switching and switch costs. Psychophysiology 2005;42(1):56–71. 10.1111/j.1469-8986.2005.00262.x 15720581

[41] Poldrack RA, Wagner AD, Prull MW, Desmond JE, Glover GH, Gabrieli JDE. Functional specialization for semantic and phonological processing in left inferior prefrontal cortex. Neuroimage 1999;10:15–35. 10.1006/nimg.1999.0441 10385578

[42] Yonelinas AP, Aly M, Wang WC, Koen JD. Recollection and familiarity: Examining controversial assumptions and new directions. Hippocampus 2010;20(11):1178–94. 10.1002/hipo.20864 20848606 PMC4251874

[43] Dunlosky J, Metcalfe J. Metacognition. Sage Publications; 2008m.

[44] Koriat A, Goldsmith M. Monitoring and control processes in the strategic regulation of memory accuracy. Psychol Rev 1996;103(3):490 8759045 10.1037/0033-295x.103.3.490

[45] Roediger HL, DeSoto KA. Confidence and memory: Assessing positive and negative correlations. Memory 2014;22(1):76–91. 10.1080/09658211.2013.795974 23721250

[46] Schacter DL, Norman KA, Koutstaal W. The cognitive neuroscience of constructive memory. Annual Review of Psychology 1998;49:289–318. 10.1146/annurev.psych.49.1.289 9496626

[47] Semlitsch HV, Anderer P, Schuster P, Presslich O. A solution for reliable and valid reduction of ocular artifacts, applied to the P300 ERP. Psychophysiology 1986;23(6):695–703. 3823345 10.1111/j.1469-8986.1986.tb00696.x

[48] Poljac E, Yeung N. Cognitive control of intentions for voluntary actions in individuals with a high level of autistic traits. Journal of Autism and Developmental Disorders 2012. 10.1007/s10803-012-1509-9 PMC3490069 22434281

[49] Elchlepp H, Lavric A, Mizon GA, Monsell S. A brain-potential study of preparation for and execution of a task-switch with stimuli that afford only the relevant task. Hum Brain Mapp 2012, 5;33(5):1137–54. 10.1002/hbm.21277 21630376 PMC6870019

[50] Mayr U, Keele SW. Changing internal constraints on action: The role of backward inhibition. Journal of Experimental Psychology: General 2000;129(1):4–26. 10756484 10.1037//0096-3445.129.1.4

[51] Longman CS, Lavric A, Munteanu C, Monsell S. Attentional inertia and delayed orienting of spatial attention in task-switching. J Exp Psychol Hum Percept Perform 2014, 8;40(4):1580–602. 10.1037/a0036552 24842065

[52] Chiu Y-C, Egner T. Distractor-relevance determines whether task-switching enhances or impairs distractor memory. Journal of Experimental Psychology: Human Perception and Performance 2016;42(1):1 10.1037/xhp0000181 26594883 PMC4688095

[53] Paller KA, Mccarthy G, Wood CC. ERPs predictive of subsequent recall and recognition performance. Biol Psychol 1988, 6;26(1–3):269–76. 3207786 10.1016/0301-0511(88)90023-3

[54] Sommer W, Schweinberger SR, Matt J. Human brain potential correlates of face encoding into memory. Electroencephalogr Clin Neurophysiol 1991, 12;79(6):457–63. 1721573 10.1016/0013-4694(91)90165-z

[55] Uncapher MR, Rugg MD. Selecting for memory? The influence of selective attention on the mnemonic binding of contextual information. Journal of Neuroscience 2009, 6 24;29(25):8270–9. 10.1523/JNEUROSCI.1043-09.2009 19553466 PMC2730727

